# Lack of evidence for retroviral infections formerly related to chronic fatigue in Spanish Fibromyalgia patients

**DOI:** 10.1186/1743-422X-10-332

**Published:** 2013-11-11

**Authors:** Elisa Oltra, María García-Escudero, Armando Vicente Mena-Durán, Vicente Monsalve, Germán Cerdá-Olmedo

**Affiliations:** 1 Cátedra Umivale en innovación e investigación en patologías del trabajo, C/Quevedo, 2, 46001 Valencia, Spain; 2 Facultad de Medicina, C/Quevedo, 2, 46001 Valencia, Spain

**Keywords:** Fibromyalgia, Chronic fatigue, Murine leukemia virus (MLV)-related virus, Xenotropic murine leukemia virus-related virus (XMRV), T-lymphotropic virus type 2 (HTLV-2)

## Abstract

**Background:**

The etiology of fibromyalgia and chronic fatigue syndrome (FM/CFS) is currently unknown. A recurrent viral infection is an attractive hypothesis repeatedly found in the literature since it would explain the persistent pain and tiredness these patients suffer from. The initial striking link of two distinct orphan retroviruses: the gamma retroviruses murine leukemia virus (MLV)-related virus and the delta retrovirus T-lymphotropic virus type 2 (HTLV-2) to chronic fatigue have not been confirmed to date.

**Results:**

Genomic DNA (gDNA) from 75 fibromyalgia patients suffering from chronic fatigue and 79 age-matched local healthy controls were screened for the presence of MLV-related and HTLV-2 related proviral sequences. The XMRV *env* gene was amplified in 20% of samples tested (24% patients/15% healthy controls). Unexpectedly, no PCR amplifications from independent gDNA preparations of the same individuals were obtained. None of the positive samples showed presence of contaminating murine sequences previously reported by other investigators, neither contained additional regions of the virus making us conclude that the initial *env* amplification came from spurious air-driven amplicon contaminants. No specific HTLV-2 sequences were obtained at any time from any of the 154 quality-controlled gDNA preparations screened.

**Conclusions:**

Previous associations between MLV-related or HTLV-2 retrovirus infection with chronic fatigue must be discarded. Thus, studies showing positive amplification of HTLV-2 sequences from chronic fatigue participants should be revised for possible undetected technical problems.

To avoid false positives of viral infection, not only extreme precautions should be taken when nested-PCR reactions are prepared and exhaustive foreign DNA contamination controls performed, but also consistent amplification of diverse regions of the virus in independent preparations from the same individual must be demanded.

The fact that our cohort of patients did not present evidence of any of the two types of retroviral infection formerly associated to chronic fatigue does not rule out the possibility that other viruses are involved in inciting or maintaining fibromyalgia and/or chronic fatigue conditions.

## Background

Fibromyalgia and Chronic fatigue Syndrome (FM/CFS) are characterized by long-lasting debilitating fatigue, often accompanied by widespread muscle pain. Its incidence is rising and ranges between 0.5-6% with high prevalence in females [1]. The high socioeconomic expense that this disease supposes is worrisome.

Currently the FM/CFS diagnosis is made solely on clinically grounds, as no biological markers associated with the disease have been found. Alteration in cytokine profiling, decreased function of natural killer (NK) cells, presence of autoantibodies and reduced response of T cells to mitogens and other specific antigens have been reported in these patients [2-4]. The observed high level of pro-inflammatory cytokines may explain some of the manifestations such as fatigue and flu-like symptoms and modify NK cells activity. Existence of RNaseL isoforms reflecting an aberrant immune system function is also well documented [5,6]. Identification of markers consistently associated with this pathology will enable clinicians to effectively diagnose FM/CFS, follow the progress of the disease, monitor the effects of therapeutic approaches and probably develop preventive programs.

Since many of the symptoms characterizing FM/CFS resemble those of infectious diseases other authors have investigated a possible viral etiology of the disease. In October 2009, Lombardi *et al*. reported the finding of a novel gammaretrovirus named xenotropic murine leukemia virus-related virus (XMRV) in about 67% (68/101) of CFS patient DNA samples prepared from peripheral blood mononuclear cell (PBMC) compared to only 3.6% (5/218) of the samples prepared from healthy controls [7]. This finding, followed by the report on the presence of MLV-related virus sequences sharing 96% of identity with the XMRV in blood of patients with CFS published in 2010 by Lo *et al.*[8] prompted us to examine whether our local population of fibromyalgia patients affected by chronic fatigue also presented evidence of murine-leukemia virus-related infection. Our initial positive amplifications of XMRV sequences coincided with numerous reports attributing XMRV amplification to murine DNA contamination of reagents [9-14], making us investigate the possibility of spurious amplifications.

A second retrovirus, namely the human T-lymphotropic virus type 2 (HTLV-2), had also been linked to chronic fatigue by a single study [15]. Contrary to XMRV screening studies, evaluation of HTLV-2 in different cohorts of FM/CFS patients has been scarce [16-18]. This latter retrovirus, closely related to the HTLV-1 virus causing adult T-cell leukemia and lymphoma and the demyelinating disease called myelopathy/Tropical spastic paraparesis (HAM/TSP) has not yet been clearly associated with any disease. Since DeFreitas *et al.* reported that 83% (10/12) of chronic fatigue patients had a positive amplification of *gag* HTLV-2 sequence compared to 0% (0/20) in matched healthy subjects we considered that a screening of HTLV-2 sequences in our patient cohort was pertinent.

In addition, antiretroviral therapy had been shown to be of limited value in controlling HTLV-2 virus expression in patients co-infected with a second retrovirus, at least for co-infections with the HIV virus [19,20]. Thus, finding out whether any of the participating patients were co-infected with a second retrovirus could be of relevance to patient care.

In this study we evaluated the presence of both retroviruses: XMRV and HTLV-2 formerly associated to chronic fatigue, and their close relatives, in a cohort of 75 Spanish fibromyalgia patients affected of chronic fatigue and 79 population-matched healthy subjects. This is, to our knowledge, the largest cohort of patients suffering from chronic fatigue evaluated for infection with the HTLV-2 virus.

## Results

### Study population

Patients’ median age was 52 yrs (range 25–76), female sex was the most prevalent (94%) and the vast majority lived in the urban area of Valencia (Spain). All patients had pain in both sides of their body as well as pain above and below their waist, with axial skeletal pain involvement. Median number of trigger points defined by the American College of Rheumatology (ACR) criteria [21] was 14 (range 11–18). Most patients (52%) were under welfare protection because of disability caused by FM. Participants had suffered from FM for a median time of 17 yrs (range 0–40). MFI average score for general fatigue of fibromyalgia patients was 18.36 ± 2.35 (range 7–20). Physical fatigue scores were more prominent than mental fatigue’s with average scores of 17.29 ± 2.81 (range 8–20) and 14.74 ± 4.32 (range 4–20) respectively.

### Inconsistent amplification of *env* XMRV sequence

**Figure 1:**
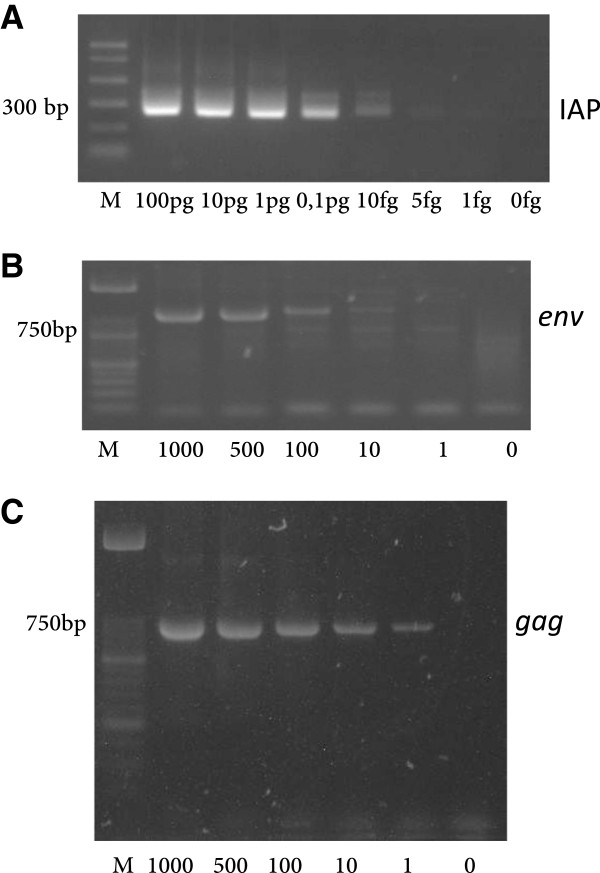
**IAP and XMRV amplification sensitivity assays. Panel A**: the indicated amounts of mouse genomic DNA were spiked into 1 μg of human gDNA and IAP sequences (236–312 bp) were amplified using previously described conditions [10]. **Panels B and C** show amplification of the *env* (973 bp) and *gag* (730 bp) genes using each outer primer set respectively, from the pcDNA3.1-VP62 construct (AIDS Research and Reference Reagent Program Cat# 11881) (number of copies 0–1000 as indicated) spiked into 1 μg of human gDNA with the outer amplification primers and conditions described in Methods. All PCR products were visualized on 2% agarose real-safe stained gels. M: molecular DNA markers correspond to PCR markers (Promega) **(panel A)** and marker XIII (Roche) **(panels B and C)**.

An initial nested PCR screening of the *env* XMRV sequence in our fibromyalgia (n = 75) and control samples (n = 79) showed close to a 20% positive amplification that corresponded to 24% (18/75) of fibromyalgia patient samples and to 15% (12/79) of the healthy control samples (Additional file 1: Figure S1, panel A and Table 1). Only once a negative control with no DNA showed positive *env* amplification. The in-house designed primer set used for the detection of XMRV and MLV-related pro-viral sequences span sequences conserved to both viruses, as described in material and methods, so that the assay would detect not only the XMRV but also related virus. However, sequencing of the PCR amplified products yielded sequences identical to the XMRV *env* sequence in all cases (data not shown). At that precise time several reports had shown that some commercial polymerases and PCR kits contained murine DNA contaminants [9-14]. In fact hybridomas were known to contain XMLV (xenotropic murine leukaemia virus) particles for long [22]. The polymerase used in our assays was not a hotstart polymerase, but our supplier could not guarantee absence of contaminant mouse DNA in the reagents. In order to discard the possibility that mouse DNA contaminants were the source of our *env* amplified sequences a sensitive PCR assay based on detection of the high-copy intracisternal A-particle (IAP) transposable elements was used [10,23]. This assay was selected for its known superior sensitivity to the mouse mitochondrial DNA PCR test which relies on the IAP high copy number in the mouse genome [10,24]. In addition, since some DNA preparing columns have been questioned to contain mouse DNA traces [25] a mock elute control consisting of DNA rehydration solution added to the master mix was included. Sensitivity assays showed positive amplification of IAP sequences up to 5 fg of mouse gDNA spiked into 1 μg of human gDNA under our amplication conditions (Figure 1A). However, no amplification proceeded in any of the samples or the negative controls confirming that neither our samples nor the reagents used contained contaminating mouse DNA (Additional file 1: Figure S1, panel B).

**Table 1:** Screening of retroviruses by nested PCR

	**Samples positive/assayed**	**XMRV/MLV**				**HTLV-2**
		***env***		***gag***		***gag***
		***Assay1***	***Assay2***	***GAG-I-F/R***	***NP116/117***	
**F**	gDNAprep1 (n = 75)	18/75	8/18^*^	0/75	0/75	0/75
	gDNAprep2 (n = 18)	ND	0/18	0/18	0/18	0/18
**C**	gDNAprep1 (n = 79)	12/79	5/12^*^	0/79	0/79	0/79
	gDNAprep2 (n = 12)	ND	0/12	0/12	0/12	0/12

The table shows the ratio between positive samples and the number of samples assayed as determined by nested PCR amplification of retroviral genes using different sets of specific primers in fibromyalgia patients (F) and healthy controls (C), as indicated. The gDNA preps2 correspond to independent preparations from frozen aliquots of the exact same participants that gave a positive amplification in the first assay (Assay1). The asterisk (*) indicates that only positive samples in the first *env* amplification (Assay 1) were tested.

### False *env* XMRV sequence amplification in blood samples due to VP62 XMRV control-derived amplicon contamination

To evaluate whether the *env* amplified sequences were product of air driven contaminants coming from the XMRV VP62 control reactions, the screening was repeated, this time using two different PCR-work stations placed in separated laboratories for the setting of sample and control reactions. Under these extreme precautions, still more than one third of the samples that showed initial *env* amplification: (8/18) patient samples and (5/12) control samples showed positive PCR amplification of *env* sequences. However, none of the 30 *env* positive samples (18 patients and 12 controls) were able to amplify *gag* sequences with any of two sets of primers formerly used to successfully amplify MLV related sequences [7,8] (Table 1)*.*

The most likely explanation for this puzzling result was that our samples were contaminated with the VP62 XMRV control first round PCR products which lack any *gag* sequence. To further confirm this was the case new genomic DNA was prepared from all the samples that had turned positive in the first round of screening.

Repetition of PCR amplification with the same *env* primers set showed negative results in all 30 samples (Additional file 1: Figure S1, panel C) allowing us to confirm that the initial amplification of *env* sequences in 20% of the samples was due to contamination by amplicons from the control reactions.

### Lack of amplification of XMRV sequences in sensitive nested PCR assays is neither due to lack of gDNA integrity nor to the presence of PCR inhibitors

To rule out the possibility that the lack of amplification of viral sequences was due to degradation of the gDNA template and/or presence of PCR inhibitors the 30 (18 patients and 12 controls) newly prepared samples and also the initial gDNA samples showing no XMRV amplification (57 patient and 67 control samples) were analyzed by gel electrophoresis and by PCR amplification of known genomic sequences (the house-keeping gene β-actin and a fragment of the human chromosome X [Genebank accession: NT_079573]). The latter was amplified with the GAPDH specific primers designed Lombardi *et al*. [7]. Which in addition to match the GAPDH mRNA sequence between nucleotides 107 and 334 corresponding to exons 1 and 2 and their corresponding location on chromosome 12, also match the human genomic X chromosome GRCh37.p10 contig sequence between positions 2499130 and 2498903. All 75 patient and the 79 control samples showed gDNA larger than 23 kb and showed positive amplification of both fragments, allowing us to conclude that lack of XMRV amplification is neither due to lack of gDNA integrity nor to the presence of PCR amplification inhibitors (Additional file 2: Figure S2). In addition, sensitivity assays of *env* and *gag* sequences showed detection of 1–10 copies from the pcDNA3.1-XMRV control spiked into 1 μg of human gDNA (Figure 1, panels B and C) indicating that lack of detection is most likely due to lack of infection.

### Lack of amplification of MLV *gag* related sequences

**Figure 2:**
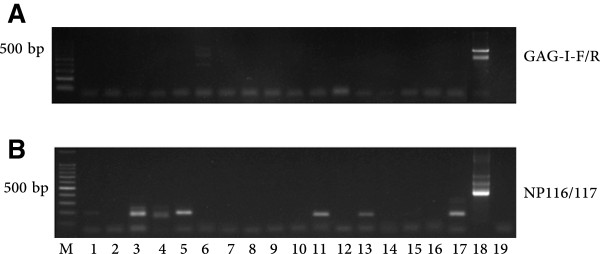
**Amplified products with *gag *primers were unspecific.** Representative nested PCR amplification of 17 gDNA samples from 9 patient samples (lanes 1–9) and 8 healthy controls (lanes 10–17) visualized on 2% agarose real-safe stained gels. Lane 18 corresponds to the VP62 XMRV positive control and lane 19 is a negative control with no DNA template. M is the 100 bp ladder marker (Promega). **Panel A** shows the products amplified with the GAG-I-F and GAG-I-R primers (410 bp) [7,44] while **Panel B** shows the products of the NP116 and NP117 primers (380 bp) [8]. All amplifications were performed under the permissive conditions described in materials and methods.

In order to determine whether the samples analyzed could contain other retroviruses partially related to the MLV virus, the nested PCR amplifications were performed at lower annealing temperatures up to 5°C below primers melting temperature (Tm), which would allow annealing to, and thus amplification of non-perfect matches. Under these permissive amplification conditions, several bands were obtained with both the GAG-I-F and GAG-I-R set and more abundantly with the NP116/NP117 set (Figure 2). No bands were obtained for the *env* set of primers under these same amplification conditions (data not shown). Although most bands had a smaller size, some of them showed a molecular weight close to the expected *gag* MLV or XMRV product size. However, sequencing of these PCR amplified fragments confirmed that all of them corresponded to unspecific amplification of human genomic sequences.

### Lack of HTLV-2 or related sequences amplification

An additional objective of the present study was to determine whether the participating fibromyalgia patients suffering of chronic fatigue were infected with the HTLV-2 retrovirus formerly related to chronic fatigue patients [15].

Using a similar nested PCR based approach used to screen XMRV and MLV-related sequences the gDNA samples of patients and controls were rescreened; this time HTLV-2 sequence specific primers under formerly described conditions [26] were used.

**Figure 3:**
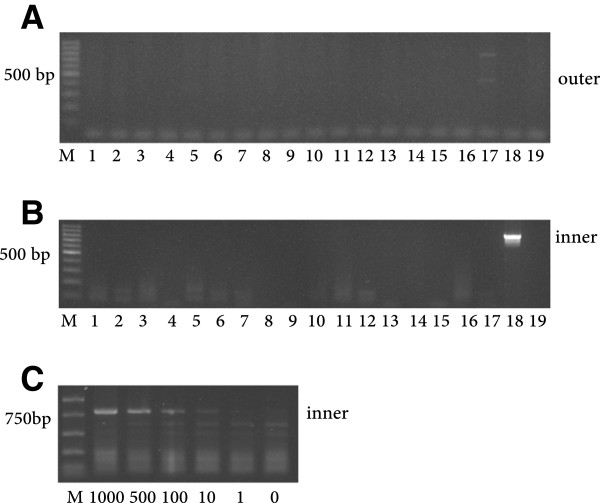
**Lack of HTLV-2 amplification.** Representative nested PCR amplification of 17 samples (10 patient and 7 healthy controls) (lanes 1–10 and 11–17 respectively) visualized on 2% agarose real-safe stained gels. **Panel A** shows the products of the first round of PCR amplification (outer primers), while **Panel B** shows the nested PCR final products (inner primers). Lane 18 corresponds to a HTLV-2 patient positive gDNA kindly provided by Drs. Treviño and Soriano [26], and lane 19 is a negative control with no DNA template. **Panel C** shows amplification of the *gag* HTLV-2 gene from the pGEMT-HTLV-2-gag construct (see Methods) (number of copies 0–1000 as indicated) spiked into 1 μg of human gDNA with the inner amplification primers and conditions described in Methods. All PCR products were visualized on 2% agarose real-safe stained gels. M: molecular DNA markers correspond to 100 bp ladder marker (Promega) **(panels A and B)** and PCR markers (Promega) **(panel C)**.

None of the fibromyalgia (n = 75) or the healthy blood donor controls (n = 79) showed amplification of a HTLV2-specific sequence (Table 1). In order to investigate whether the samples contained other retroviral sequences related to HTLV-2 the screening was repeated using similar permissive amplification conditions previously mentioned. Under these low temperature conditions no final amplification product was obtained either (Figure 3, panel B). Unexpectedly, 2 patient samples showed amplification in the first round of PCR, one of the bands had a size close to the expected product (802 bp). The other had an approximate size of 450 bp (Figure 3, panel A; sample 17). Subcloning and sequencing of either band revealed that none of them contained viral sequences. Again, sensitivity assays using subcloned HTLV-2 *gag* sequences spiked into 1 μg of human gDNA showed that our nested-PCR assay detection threshold was 1–10 viral copies (Figure 3, panel C). Thus, neither the FM/CFS samples nor the healthy controls presented any evidence of infection by viruses related to HTLV-2.

## Discussion

This is the first report to our knowledge that evaluates the presence of the both families of retrovirus formerly associated with chronic fatigue: the gamma retroviruses MLV-related and the delta retrovirus HTLV-2 in a cohort of patients suffering from fibromyalgia and chronic fatigue.

At present enough evidence exists to support the inexistence of a natural form of the XMRV virus in humans [14,27,28]. However, since the XMRV is competent to infect and replicate within human cells [29] and some of its relatives: the xenotropic-MLV (X-MLV) viruses can infect non-human primates and other species causing disease [22,30], the possibility of a human xeno-infection by an MLV-related virus remains.

Opposite to the actively studied relationship of the XMRV to chronic fatigue, the association or non-association of the orphan HTLV-2 retrovirus to the disease has not been clarified. The initial study which used PCR amplification of HTLV-2 proviral sequences followed by probe hybridization assays to find an association of HTLV-2 infection to CFS was followed by only a few negative studies [16-18]. Even though this technique is highly specific and sensitive, amplification by nested PCR may be superior when detection of low levels of infection and/or virus variants is pursued. In addition none of the three negative studies that followed the initial one included more than 30 patient blood samples [16-18], and therefore further investigation including larger cohorts of patients was at need.

This, to our knowledge, constitutes the study with the largest number of patients (n = 75) of fibromyalgia suffering from chronic fatigue examined for infection by HTLV-2. None of the samples assayed showed specific amplification of HTLV-2 or related sequences at any time making us conclude a non-association between HTLV-2 retroviral infection and the chronic fatigue condition of our patients. As we already proposed [31] HTLV-2 association with CFS reported by DeFreitas [15] may also be caused by undetected technical problems like it occurred with XMRV and CFS [7].

It is also interesting to note that the molecular weight of one of the two bands obtained in our first PCR amplification coincides with the endogenous unspecific 440 bp *gag* band obtained by Gow *et al*. [17] in the first round PCR step. However, different to them, we could only observe it in 2/154 samples assayed (Figure 3, panel A) and therefore we cannot coin it as endogenous.

Contrary to the negative results obtained in the HTLV-2 screen, our initial screening analysis of XMRV and MLV-related sequences yielded a moderate percentage of positives 20% (30/154) with a slight predominance on the patient subgroup (24% positive patients versus 15% positive healthy controls).

The lack of sequence diversity of our PCR amplified products with respect to the american VP62 XMRV isolate we were using as the positive control made us suspect our samples could be contaminated with a specific template. Even though our lab does not harbor mice work and nor mouse-derived or XMRV-infected cell lines are maintained we proceeded to perform a sensitive IAP test to evaluate whether the *env* positive amplifications could come from mouse DNA contamination in any of the components used. Different to other researchers we could not find any presence of murine DNA.

If the obtained amplified sequences were really derived from our participant blood samples we should be able not only to reproduce the obtained results from an independently prepared sample from the same patient but also to detect other parts of the viral genome. The fact that the initial positive amplification of the XMRV *env* sequence could not be reproduced in an independent extraction of gDNA prepared from frozen aliquots of the same exact samples, added to the complete lack of amplification of other regions of the viral genome, specifically the *gag* sequence, made us conclude that the initial amplification should have come from fortuitous air driven spreading of *env* specific amplicons, most likely product of the first round of PCR of our VP62 XMRV positive control. This conclusion points out the extreme importance of independently confirming positive results when using highly sensitive assays.

In addition to the two initial reports by Lombardi *et al*. and Lo *et al*. which showed a striking association between the presence of XMRV or MLV-related sequences and a chronic fatigue condition, both of which are currently retracted, some other groups of researchers have also reported positive amplification of murine gamma retrovirus sequences in human samples. Most amplifications have been attributed to sample contamination with mouse DNA [9-14], other, confirming lack of contaminant mouse DNA, have not been able to find an alternative explanation, but as in our study, their positive results could never be confirmed for a second viral gene in the same sample [32-34]. It is therefore possible that their inconsistent amplifications may come from spurious amplicon contamination similar to the ones we detected. Alternatively, inconsistent PCR amplifications could come from extremely scarce target sequence presence. However, being that most of the studies finding inconsistent viral sequence amplifications, including ours; used 0.5-1 μg of gDNA template in their assays [32,35] it is very unlikely this could be the case. Spiking amplification assays determined 1–10 copies to be the lower threshold limit for successful nested PCR amplification in the context of 1 μg of human gDNA, coinciding with previous reports [35], and since each somatic cell has 6.16 pg of DNA, less than 1 in 3 × 10^5^ cells would need to be infected for such a scenario to take place.

Although, great efforts had been directed to look for MLV related infection in CFS patients only a few [33,36-38] included fibromyalgia patients in the studied groups, and only one of them included a larger cohort of patients that our study [33].

To ensure that our testing would not miss genetically diverse XMRV, MLV or HTLV-2 strains we decreased the annealing temperature to allow missprimed amplification. Even though some bands were amplified under these non-stringent conditions none of them contained viral sequences, most probably indicating absence of related infections in our patient and control participants.

Because negative PCR results would be obtained from defective samples, we considered necessary to rigorously perform controls to guarantee that lack of amplification was not due to degraded or impure DNA preps. Even though amplification of house-keeping genes which are present at a frequency of 2 copies per cell does not guarantee amplification of less abundant templates, at least rules out the presence of potent inhibitors of PCR amplification.

It was noticed that the GAPDH primer set used in Figure 1 of the Lombardi *et al*. study [7] amplifies a human non-related genomic sequence in chromosome X of a similar size to expected GAPDH RT-PCR product (228 vs 227 bp) and therefore careful interpretation of the results obtained with it should be taken. In our study it served the purpose of detecting amplification of a region of the genome (Additional file 1: Figure S1, panel B) to evaluate the quality of the gDNA preps screened.

## Conclusions

No evidence of infection by any of the two retroviruses formerly associated to chronic fatigue syndrome: the XMRV and the HTLV-2 or their close relatives was found in any of the 154 tested samples.

The *env* positive amplifications obtained in our study (20% of the samples) derived from a specific source: amplicons corresponding to the products of our positive control sample: the VP62 isolate of the XMRV. It is, therefore, our recommendation that in addition to rigorous contamination checkups and restricted access of positive controls to sample working areas; independent amplification of more than one gene of the virus is confirmed from more than one independent sample preparation from a particular individual in order to diagnose infection by the sensitive nested PCR method. We also recommend the inclusion of controls to guarantee the quality of the gDNA template evaluated especially when all results obtained are negative.

The negative results reported in this and other studies do not allow ruling out the possibility of a viral or a set of viral infections to be the origin for the fibromyalgia and/or chronic fatigue syndrome. Low cost high throughput, deep sequencing assays are powerful tools to identify all viral species commonly infecting humans (the human virome) and also to allow a comprehensive understanding of viral variation and evolution during replication and transmission events. The use of these techniques in metagenomic studies is critical as we aim to understand how the human virome affects long-term human health, immunity, and response to coinfections [39].

## Methods

### Patients and healthy controls

From January 2011 to April 2011, 75 patients and 79 healthy participants individually matched by age (range +/− 5 yrs) were recruited for this study. All 75 patients came from a single Institution, Catholic University Medical School in Valencia, Spain and meet the criteria for fibromyalgia diagnosis according to the American College of Rheumatology’s (1990) [21]. Healthy matched controls were regular blood donors from the Valencian Community Blood Bank. A single sample of whole blood was obtained from each individual after signing an informed consent form. Each participant underwent a thorough clinical interview to assess clinical criteria and severity of fibromyalgia using standardized Fibromyalgia Impact Questionnaire (FIQ) case report forms [40,41]. For fatigue assessment the Multi-fatigue inventory MFI was used [42]. Those with a score ≥ well population medians on the general fatigue or reduced activity scales of the MFI were considered to meet fatigue criteria of the 1994 international case definition. Patient data were entered into a computer database (Microsoft Access 2003, Redmond, WA: Microsoft Corp). The study was approved by the Hospital de la Plana de Vila-Real (Castellón, Spain) CEIC. Written informed consent was obtained from patients for the publication of this report and any accompanying images.

### Specimen collection, processing and storage

Approximately 20 ml of blood per patient was collected in 2 Vacutainer tubes containing 170 IU of lithium heparin (Becton Dickinson BD 365725) were hand-carried at RT and processed within 2 h. After taking 2.4 ml of fresh blood aliquoted into 2 vials: one to be freshly processed for genomic DNA (gDNA) and the other to be kept at −80°C, the remaining sample was subjected to Ficoll based gradient separation for the isolation of PBMCs.

### Isolation of PBMCs

Samples diluted at 1:1 (v/v) ratio in phosphate-buffered saline solution (PBS) were layered on top of 1 vol of Ficoll-Paque Premium (GE Healthcare) and subjected to density centrifugation at 20°C at 500 g for 30 min (brakes off). The PBMC layer was removed, washed with PBS and centrifuged (brakes on). The isolated PBMC pellets were resuspended in 1 vol of red blood cell lysis buffer (155 mM NH4Cl, 10 mM NaHCO3, pH 7.4, 0.1 mM EDTA), kept on ice for 5 min, and centrifuged (20°C, 500 g, 10 min), as previously described [43]. The PBMC were washed with PBS and centrifuged again. The pellets were resuspended in freezing medium (90% FBS, 10% DMSO) concentration adjusted to 10^7^mononuclear cells/ml aliquoted and frozen at −150°C until use.

### Isolation of genomic DNA

The Wizard Genomic DNA Purification kit (Promega) was used to isolate human gDNA either from 600 μl of fresh whole blood or from 600 μl of pre-frozen whole blood or pre-frozen PBMCs for samples that needed a second preparation of gDNA (*env* positive samples), following manufacturer’s recommendations. The same kit was used to isolate mouse gDNA from 600 μl of mouse tail fresh whole blood under equivalent conditions. gDNA yield was measured by spectroscopic absorption at 260 nm in a nanodrop 2000c (Thermo Scientific) and concentration was adjusted to 200 ng/μl in TE buffer. gDNA stocks were kept at 4°C in tightly capped tubes to prevent gDNA breakage by freeze-thaw cycles.

### Nested PCR amplifications

The nested PCR for the *env* and *gag* genes were performed according to the protocols described previously [7,8,44] with some modifications. For the first round of PCR, the amplification reaction performed in a final volume of 50 μl contained 1 μg of gDNA, 1× Green Go Taq Flexi buffer, 2,5 mM MgCl_2_, 0,2 mM dNTPs, 1 pmol/μl of each primer and 2,5 units of Go Taq Flexi DNA polymerase (Promega) and 5% DMSO. The cycles were 94°C 5 min, (1 min at 94°C, 30 sec at 55°C and 1 min at 72°C) ×40 cycles and 10 min at 72°C. For the second round PCR the amplification reaction was the same except that contained 1/10 vol of the first round as the target sequence to be amplified, the Tm used in the annealing step range from 50 to 60°C depending on the primer set used; the number of cycles was 45 in all cases. As PCR amplification positive control a pcDNA3.1 construct containing the XMRV VP62 cDNA (Cat# 11881) provided by the AIDS Research and Reference Reagent Program was used. Negative control contained all components in the master mix but no DNA. The conditions used for the amplification of HTLV-2 sequences were the same described else were [26] except that the annealing temperature of the second round was lowered to 55°C to achieve permissive amplification conditions. Positive controls for HTLV-2 amplification consisted of gDNA isolated from a HTLV-2 positive patient which was kindly provided by Drs. Soriano and Treviño at the Hospital Carlos III (Madrid, Spain) or a self-made construct using a PCR amplified HTLV-2 fragment from the first round (outer primers) subcloned in the pGEM-T Easy vector (Promega).

#### For detection of XMRV and MLV-related viral sequences

Primers to amplify conserved *env* sequences were self-designed upon alignment of the Xenotropic MuLV-related virus VP62 complete genome [Genbank: DQ399707] with the Murine Leukemia Virus MCF1233 complete genome [Genbank: U13766]. For the first round PCR the primers selected were XMRV-*env*-outer-F 5′-TGTGAGACCACTGGACAGGC-3′and XMRV-*env*-outer-R 5′-GTAAGTCCTCCCAACAGCAG-3′ and for the nested PCR the primers XMRV-*env*-inner-F 5′- ACGCGGGTAAAAGGGCCAGC-3′and XMRV-*env*-inner-R 5′-AAGCCCAAATGGTCCCGGCG-3′ were used.

The primers to amplify *gag* XMRV sequences were the same used by Urisman *et al*. and Lombardi *et al*. to detect 413-bp XMRV *gag* sequences in prostate cancer and CFS patients, 419 F and 1154R (outer) and GAG-I-F and GAG-I-R respectively [7,44]. As inner primers the NP116 and NP117, designed by Lo *et al.*[8] to anneal to highly conserved sequences in different MLV-like viruses and XMRVs, were also used.

#### For detection of HTLV-2 sequences

The outer primers were: 5′-CTAGCCTCCCAAGCCAGCCACC-3′ as the forward primer and 5′-CCAGTGGTGGGTTGATAGCCC-3′ as the reverse. The inner primers were: 5′-CGAGTCATCGACCCAAAAGGTC-3′ and 5′-GGAGTTGGGGAAAGCCCGTGG-3′forward and reverse, respectively, for detection of an 802 bp LTR fragment of the HTLV-2 2b southern Europe subtype, formerly described by Toro *et al.*[26].

### PCR amplifications

#### For detection of IAP sequences

The primers IAP-F 5′-ATAATCTGCGCATGAGCCAAGG-3′ and IAP-R 5′-AGGAAGAACACCACAGACCAGA-3′ and previously described amplification conditions were used [10].

#### For detection of house-keeping gene sequences

The primers for the human genomic X chromosome GRCh37.p10 contig sequence between positions 2499130 and 2498903 were described elsewere [7] as GAPDH forward - 5′ GGAAGGTGAAGGTCGGAGTC 3′ and reverse - 5′ GGAAGATGGTGATGGGATTTC 3′. The house designed β-actin primer sequences were: 5′ATATCGCCGCGCTCGTCGTC 3′(forward) and 5′GAGCCACACGCAGCTCATTG 3′ (reverse). 50–100 ng genomic DNA were used under the standard PCR amplification conditions described, 3 minutes at 95°C followed by 30 cycles of 95°C for 30 seconds, 55°C for 30 seconds and 72°C for 30 seconds, followed by 1 cycle of 72°C for 2 minutes.

All assays were optimized to achieve the highest sensitivity in detecting the target sequences up to 110 copies of either target plasmid control sequence spiked in one μg of human genomic DNA before start which coincided with previous reports [32,35]. One μg of human DNA was used as input for the PCR tests and all PCR products were resolved by 2% agarose gel electrophoresis and visualized by real safe-staining.

### Subcloning, sequencing of PCR amplified fragments and BLAST alignments

The pGEM-T Easy (Promega) vector was used to subclone gel purified (GenElute™ Agarose Spin Columns, Sigma) PCR amplified fragments by TA cloning according to manufacturer protocols. Sequencing was performed at the sequencing facility: Unidad de Genómica del Servicio Central de Soporte a la Investigación Experimental (SCSIE) de la Universidad de Valencia, using the T7 forward and SP6 reverse primers under standard conditions. Sequences obtained were aligned to the non-repetitive sequences at the Genbank database using the Basic local alignment search tool at the NCBI [45].

## Abbreviations

FM/CFS: Fibromyalgia/chronic fatigue syndrome; NK: Natural killer; PBMCs: Peripheral blood mononuclear cells; FIQ: Fibromyalgia Impact Questionnaire; MFI: Multi-fatigue inventory; MLV: Murine leukemia virus; XMRV: Xenotropic murine leukemia virus-related virus; HTLV-2: T-lymphotropic virus type 2; IAP: Intracisternal A- type particle; GAPDH: Glyceraldehyde-3-phosphate dehydrogenase.

## Competing interests

The authors declare they have no competing interests.

## Authors’ contributions

EO, AMD, VM and GCO conceived and designed the study. VM, AMD, MGE and GCO confirmed patient diagnostic and collected data on participating patients. EO, MGE and AMD performed specimen testing and data analysis. EO and AMD wrote the manuscript. All authors read and approved the final manuscript.

## Supplementary Material

Additional file 1: Figure S1 *Env* positive samples were not contaminated with mouse DNA. Panel A: representative result of the first round of XMRV *env* screening by nested PCR (602 bp). Lanes 1–12 contained gDNA from 7 patient samples (lanes 1–7) and 5 healthy controls (lanes 8–12). Positive and negative controls corresponding to pcDNA3.1-VP62 (AIDS Research and Reference Reagent Program Cat# 11881) and no gDNA, are shown in lanes 14 and 13 respectively. Panel B: amplification of IAP mouse sequences (236–312 bp) from 17 *env* positive samples (11 patients and 6 controls) (lanes 1–11 and 12–17 respectively). Lane 18 shows amplification from mouse genomic DNA (positive control) while lane 19 shows amplification of a mock gDNA elute (negative control). Panel C: *env* screening by nested PCR of the same *env* positive samples shown in panel B now using new independent preparations of gDNA. All PCR products were visualized on 2% agarose real-safe stained gels. M: 100 bp ladder marker (Promega). Click here for file

Additional file 2: Figure S2 gDNA preparations from PBMCs were intact and pure. Panel A: representative gDNA from 9 patient samples (lanes 1–9) and 9 healthy controls (lanes 10–18) (0.5 μg/lane) visualized in a 1% real-safe stained agarose gel. Panel B: PCR products amplified from the same samples with either the previously described GAPDH primers (227 bp) [7] (upper) or the in-house designed β-actin set (lower) (416 bp); lane 19 shows a negative control with no DNA. All samples were visualized in 2% real-safe stained agarose gels. M: lambda phage *HindIII* DNA marker (Biotools) (panel A); M: 100 bp ladder marker (Promega)(panel B). Click here for file
