# Supplementary figures and images for: Lack of evidence for retroviral infections formerly related to chronic fatigue in Spanish Fibromyalgia patients
Source: Virol J. 2013 Nov 11;10:332. doi: 10.1186/1743-422X-10-332 (PMC4226024; doi:10.1186/1743-422X-10-332)

## Slide 1
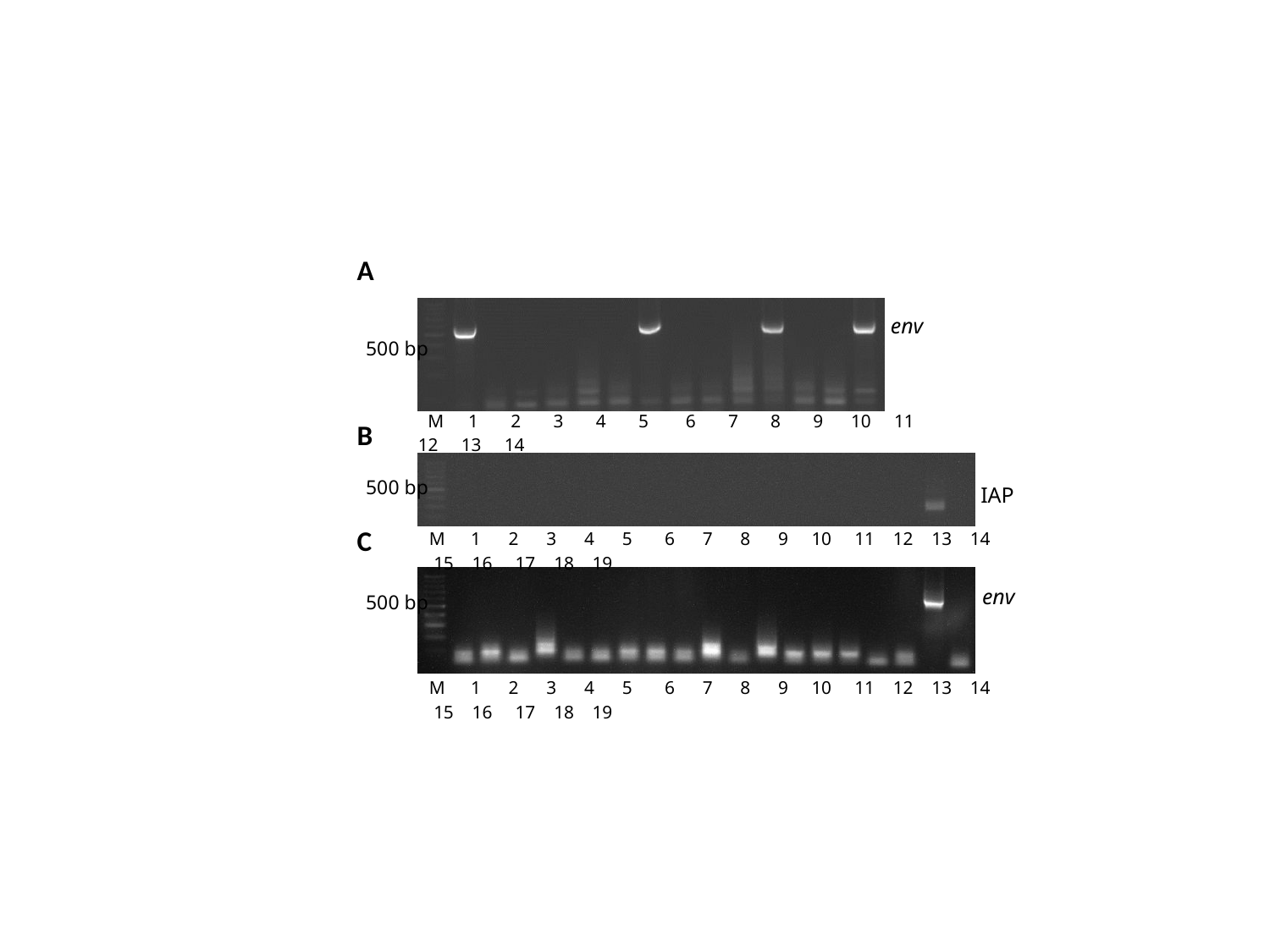

A
env
500 bp
 M 1 2 3 4 5 6 7 8 9 10 11 12 13 14
B
500 bp
IAP
C
 env
500 bp
 M 1 2 3 4 5 6 7 8 9 10 11 12 13 14 15 16 17 18 19
 M 1 2 3 4 5 6 7 8 9 10 11 12 13 14 15 16 17 18 19

Supplement: Additional file 1: Figure S1 — Env positive samples were not contaminated with mouse DNA. Panel A: representative result of the first round of XMRV env screening by nested PCR (602 bp). Lanes 1–12 contained gDNA from 7 patient samples (lanes 1–7) and 5 healthy controls (lanes 8–12). Positive and negative controls corresponding to pcDNA3.1-VP62 (AIDS Research and Reference Reagent Program Cat# 11881) and no gDNA, are shown in lanes 14 and 13 respectively. Panel B: amplification of IAP mouse sequences (236–312 bp) from 17 env positive samples (11 patients and 6 controls) (lanes 1–11 and 12–17 respectively). Lane 18 shows amplification from mouse genomic DNA (positive control) while lane 19 shows amplification of a mock gDNA elute (negative control). Panel C: env screening by nested PCR of the same env positive samples shown in panel B now using new independent preparations of gDNA. All PCR products were visualized on 2% agarose real-safe stained gels. M: 100 bp ladder marker (Promega). [file 1743-422X-10-332-S1.pptx]

## Slide 1
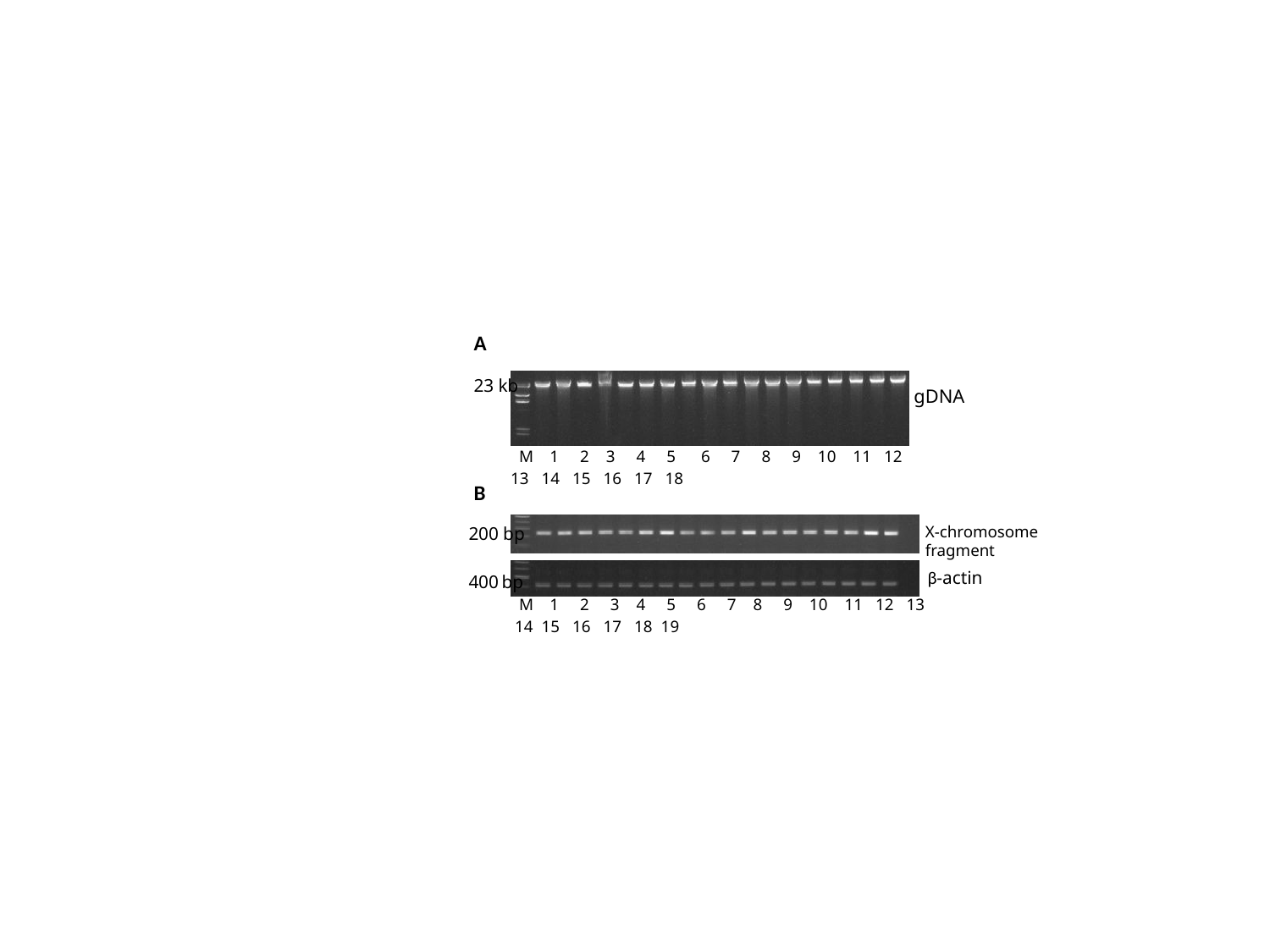

A
23 kb
gDNA
 M 1 2 3 4 5 6 7 8 9 10 11 12 13 14 15 16 17 18
B
X-chromosome fragment
200 bp
β-actin
400 bp
 M 1 2 3 4 5 6 7 8 9 10 11 12 13 14 15 16 17 18 19

Supplement: Additional file 2: Figure S2 — gDNA preparations from PBMCs were intact and pure. Panel A: representative gDNA from 9 patient samples (lanes 1–9) and 9 healthy controls (lanes 10–18) (0.5 μg/lane) visualized in a 1% real-safe stained agarose gel. Panel B: PCR products amplified from the same samples with either the previously described GAPDH primers (227 bp) [7] (upper) or the in-house designed β-actin set (lower) (416 bp); lane 19 shows a negative control with no DNA. All samples were visualized in 2% real-safe stained agarose gels. M: lambda phage HindIII DNA marker (Biotools) (panel A); M: 100 bp ladder marker (Promega)(panel B). [file 1743-422X-10-332-S2.pptx]
